# Thyroglobulin as a Functional Biomarker of Iodine Status in a Cohort Study of Pregnant Women in the United Kingdom

**DOI:** 10.1089/thy.2016.0322

**Published:** 2017-03-01

**Authors:** Sarah C. Bath, Victor J.M. Pop, Victoria L. Furmidge-Owen, Maarten A.C. Broeren, Margaret P. Rayman

**Affiliations:** ^1^ Department of Nutritional Sciences, Faculty of Health and Medical Sciences, University of Surrey, Guildford, United Kingdom.; ^2^ Department of Primary Care, University of Tilburg, Tilburg, The Netherlands.; ^3^ Danone Nutricia Early Life Nutrition, Trowbridge, United Kingdom.; ^4^ Department of Clinical Chemistry, Máxima Medical Center, Veldhoven, The Netherlands.

**Keywords:** iodine, thyroglobulin, pregnancy, thyroid, nutrition

## Abstract

***Background:*** Though iodine deficiency in pregnancy is a matter of public-health concern, a functional measure of iodine status is lacking. The thyroid-specific protein thyroglobulin (Tg), which reflects thyroid size, has shown promise as a functional measure in studies of children and adults, but data in pregnancy are sparse. In a cohort of mildly to moderately iodine-deficient pregnant women, this study aimed to explore whether serum Tg is a sensitive functional biomarker of iodine status and to examine longitudinal change in Tg with gestational age.

***Method:*** A total of 230 pregnant women were recruited at an antenatal clinic at 12 weeks of gestation to the Selenium in PRegnancy INTervention study, in Oxford, United Kingdom. Repeated measures of urinary iodine-to-creatinine ratio, serum thyrotropin (TSH), and Tg at 12, 20, and 35 weeks of gestation were made. Women were dichotomized by their iodine-to-creatinine ratio (<150 or ≥150 μg/g) to group them broadly as iodine deficient or iodine sufficient. Women with thyroid antibodies were excluded; data and samples were available for 191 women.

***Results:*** Median Tg concentrations were 21, 19, and 23 μg/L in the first, second, and third trimesters, respectively. In a linear mixed model, controlling for confounders, Tg was higher in the <150 μg/g group than it was in the ≥150 μg/g group (*p* < 0.001) but there was no difference in TSH (*p* = 0.27). Gestational week modified the effect of iodine status on TSH (*p* = 0.01) and Tg (*p* = 0.012); Tg did not increase with gestational week in the ≥150 μg/g group, but it did in the <150 μg/g group, and TSH increased more steeply in the <150 μg/g group.

***Conclusions:*** Low iodine status (<150 μg/g) in pregnancy is associated with higher serum Tg, suggesting that the thyroid is hyperstimulated by iodine deficiency, which causes it to enlarge. Tg is a more sensitive biomarker of iodine status in pregnancy than is TSH.

## Introduction

An adequate dietary supply of iodine is essential for the production of the thyroid hormones thyroxine (T4) and triiodothyronine (T3). Hence, the recent recognition that the iodine status of teenage schoolgirls (1), women of child-bearing age (2,3), and pregnant women (4–6) in the United Kingdom appears to be suboptimal has raised concern. The finding of an association between the mild-to-moderate degree of iodine deficiency found in British pregnant women and lower IQ and reading scores in their eight- to nine-year-old children (7) has increased that concern.

Iodine status is commonly assessed by measurement of urinary iodine concentration (UIC) from a spot-urine sample (8). However, UIC is not a functional biomarker of iodine status; it only reflects recent intake (past 24–48 h) and may therefore not be representative of usual intake in an individual. Although the thyroid hormones (T3 and T4) and thyrotropin (TSH) are functional measures, they are not sensitive markers of iodine status, as values can remain in the normal reference range in individuals with suboptimal iodine intake because of tight homeostatic regulation (9). By contrast, the thyroid-specific protein thyroglobulin (Tg) shows promise as a functional biomarker of iodine status that better reflects long-term iodine intake (weeks or months) (8,9). Serum Tg concentration is considered to reflect thyroid volume in both iodine-deficient and iodine-excessive settings (10). In iodine deficiency, high Tg concentration results from TSH stimulation of the thyroid, leading to thyroid enlargement (9). Antibodies to Tg (TgAb) can interfere with Tg measurement, resulting in either a higher Tg concentration when measured by radioimmunometric assay (RIA) or a lower concentration when measured by an immunometric assay (11). TgAb therefore need to be measured concurrently so that those individuals who are TgAb-positive can be excluded from analysis.

While studies have explored the relationship between iodine status and serum Tg in both adults and children (10,12), there is little exploration of this relationship in pregnancy (9), especially with a repeated-measure study design. Furthermore, there are no Tg data from pregnant women in the United Kingdom—a region of mild-to-moderate iodine deficiency. This study therefore aimed to investigate the relationship between iodine status (as measured by the iodine-to-creatinine ratio) and serum Tg concentration in a cohort of British pregnant women to test whether Tg might be a useful functional biomarker of low iodine status and to understand the change in Tg concentration during pregnancy under conditions of mild-to-moderate iodine deficiency.

The hypotheses of this study were that iodine status would be negatively associated with serum Tg and that the association would be stronger than with serum TSH. Furthermore, it was hypothesized that the profile of TSH and Tg throughout pregnancy would differ between those classified as iodine deficient and iodine sufficient. A lag effect was anticipated such that TSH would increase more steeply in iodine-deficient women as pregnancy advanced and stores of iodine became depleted.

## Materials and Methods

This study used samples and data collected as part of the Selenium in Pregnancy INTervention (SPRINT) study, a double-blind placebo-controlled randomized trial (ISRCTN37927591) that investigated the effect of selenium supplementation on markers of risk of pre-eclampsia. Two hundred and thirty primiparous women (sample size calculated to detect differences in biological markers of pre-eclampsia) were recruited when attending for an ultrasound scan at 12–14 weeks of gestation at the John Radcliffe Hospital (Oxford, United Kingdom) between July 2009 and June 2011. Relevant exclusion criteria were current smoking, being on thyroid medication, and taking a selenium-containing supplement. As most prenatal supplements contain both iodine and selenium, this exclusion criterion meant that very few women were taking an iodine-containing supplement (13). One woman was recruited in error, as she was taking levothyroxine and was therefore excluded. Full details of the SPRINT study have already been reported (14).

This study was conducted according to the guidelines laid down in the Declaration of Helsinki, and all procedures involving human subjects were approved by the Milton Keynes Research Ethics Committee (REC ref no. 08/H0603/46). A non-substantial amendment for additional laboratory measurements in stored samples was approved by NRES Committee South Central Berkshire (July 27, 2011). Written informed consent was obtained from all subjects.

### Procedures

Blood and urine samples were collected at approximately 12, 20, and 35 weeks of gestation. TSH, free T4 (fT4), and thyroid peroxidase antibodies (TPOAb) were measured in serum samples using a Modular Analytics E170 analyzer (Roche Diagnostics, Mannheim, Germany) at the Department of Clinical Chemistry, Máxima Medical Center (Veldhoven, The Netherlands). Tg and TgAb were measured using an electrochemiluminescence immunoassay on a Cobas e601 analyzer (Roche Diagnostics) at the Máxima Medical Center. The serum Tg assay was calibrated against the Certified Reference Material for human Tg (CRM-457). TPOAb concentrations >35 IU/mL and TgAb >115 KIU/L (manufacturer's cutoff) were considered as positive for TPOAb and TgAb, respectively. Women were defined as thyroid-antibody positive if they were positive for TPOAb and/or TgAb. The use of trimester- and method-specific reference ranges from an iodine-sufficient and thyroid-antibody negative population are recommended for thyroid function tests in pregnancy (15). However, no method-specific (i.e., Roche) reference ranges exist in the literature (16). Trimester-specific reference ranges for TSH and fT4 derived from women without thyroid autoimmunity from an iodine-sufficient region were therefore used (17). The reference ranges and the definitions of overt hypothyroidism, hyperthyroidism, subclinical hypothyroidism, and isolated hypothyroxinemia are reported in our previous study that had the same participants as the current study (18). For assessment of Tg, a cutoff of 40 μg/L was used to indicate high Tg concentration based on previous research in adults and children (9).

Measurement of urinary iodine and creatinine concentration was carried out at the Trace Element Unit, Southampton General Hospital, as previously described (13). Briefly, iodine was measured using a dynamic reaction cell inductively coupled plasma mass spectrometry (ICP-MS), and certified reference materials were used to ascertain the accuracy of the method. Creatinine was measured using the UniCel DxC Synchron Clinical System Analyzer by the Jaffe rate method.

The iodine-to-creatinine ratio was used in preference to the UIC, as SPRINT women were requested to attend the hospital with a full bladder for their ultrasound scan, and as a result, some urine samples were very dilute; use of the UIC would have overstated iodine deficiency in the cohort (13).

Two hundred and nineteen women (95.6% of the cohort) completed a Food Frequency Questionnaire (FFQ) at approximately 12 weeks of gestation (13), and data on milk intake from the FFQ were used to explore the association with serum Tg concentration. Our previous study in this cohort found an association between milk intake and the urinary iodine-to-creatinine ratio (13).

### Statistical analysis

As the selenium intervention had no effect on urinary iodine-to-creatinine ratio (13), TSH, or fT4 in antibody-negative women in the trial (18), data were pooled to analyze the women as one group, regardless of intervention.

There is a known effect of autoimmune thyroid disease on TSH (19), and TgAbs can interfere with the interpretation of Tg analysis (9). Therefore, women who were positive for TPOAb and/or TgAb at 12 weeks of gestation (*n* = 34; 14.9%) and those with overt thyroid disease (one woman [0.4%] had overt hyperthyroidism at 12 weeks of gestation) were excluded in order to describe more accurately the relationship between iodine status and thyroid function. Two additional women with a urinary iodine-to-creatinine ratio >700 μg/g who were clear outliers (i.e., values suggestive of excessive iodine intake) were excluded, and one woman with transient subclinical hyperthyroidism at 12 weeks was also excluded, as inclusion prevented convergence of the linear mixed models. This left 191 women for statistical analysis.

Women were dichotomized on the basis of maternal urinary iodine-to-creatinine ratio as ≥150 μg/g (i.e., sufficient) or <150 μg/g (i.e., deficient), as in our previous study (7). The groups were further divided by iodine-to-creatinine ratio as <100, 100–149, 150–249, and ≥250 μg/g, based on methodology in a Belgian study (20).

Normally distributed data (fT4) are reported as mean (standard deviation [*SD*]). Non–normally distributed data (UIC, iodine-to-creatinine ratio, TSH, and Tg) were transformed using the natural logarithm. TSH and Tg values are reported as geometric means and confidence intervals [CI] by back-transformation into the original units. The study compared (log) Tg concentration between antibody-positive and antibody-negative women, and between categories of milk consumption using *t*-tests and one-way analysis of variance, respectively. The chi-square test was used for comparison of categories of milk intake and percentage of women with Tg >40 μg/L.

A linear mixed model was used to maximize the utilizable data, as some subjects had missing data. The predictors of TSH and Tg were explored by building models that included variables that are known to affect the concentration of both (20). These variables were iodine-to-creatinine ratio (<150 vs. ≥150 μg/g), gestational week, maternal age (years), smoking status (ex-smoker vs. non-smoker), body mass index (BMI) at 12 weeks of gestation (kg/m^2^), and ethnicity (Caucasian or other). Season (summer or winter) was also included as a time-varying confounder to account for the underlying change in season with progress of gestation, as has been done previously (13). The model included random effects, with random coefficients at the subject level (i.e., intercept and gestational week). A model was constructed with an interaction term between the iodine variable and gestational week, which allowed us to explore our hypothesis that the effect of gestation would differ according to the iodine status of the individual. Interactions were tested between the iodine variable and other confounders, but these were not statistically significant. The standardized residuals at levels 1 (within-subject) and 2 (between-subjects) were visually assessed for normality. The multivariable-adjusted geometric mean ratios of TSH and Tg were estimated.

fT4 concentration was not modeled because the model would not converge if the same linear mixed model was applied as for the Tg and TSH variables (gestational week could not be included as a random effect). It was therefore not possible to evaluate the predictors of fT4 in the same way as TSH and Tg, as the models were not comparable. Furthermore, there are known problems with fT4 measurement in pregnancy that suggest that the measure may not be reliable (21).

Statistical analysis was conducted using IBM SPSS Statistics for Windows v21.0 (IBM Corp., Armonk, NY). Statistical significance was set at 5%.

## Results

At week 12 of gestation, 25 (11%) women were TPOAb-positive, 22 (9.9%) were TgAb-positive, of whom nine (3.9%) had isolated TgAb positivity (i.e., not TPOAb-positive). Geometric mean Tg concentration was lower in the 34 (14.9%) women who were positive for TgAb and/or TPOAb in the first (16 vs. 21; *p* = 0.17), second (13 vs. 18; *p* = 0.12), and third trimesters (13 vs. 21; *p* = 0.02). These women were excluded from further analysis.

The demographic data for the whole cohort of 230 women have been reported previously (14). In these 191 women, the mean (*SD*) age was 30.6 (4.2) years, the mean BMI was 24.4 (4.1) kg/m^2^, and 92.1% were Caucasian. The group was classified as mildly-to-moderately iodine deficient in all trimesters, as previously reported (13) (Table 1). TSH increased and fT4 concentration decreased over the course of pregnancy (Table 1).

**Table 1.** Thyroid Function Parameters and Urinary Iodine Concentrations in Pregnant Women Negative for TPOAb and/or TgAb Without Overt Thyroid Disease

	*First trimester*	n	*Second trimester*	n	*Third trimester*	n
Gestational week of sample^a,b^	12 (9, 14)	190	20 (17, 23)	185	35 (30, 36)	178
Iodine concentration (μg/L)^a,b^	39.4 (23.3, 84.4)	190	55.4 (32.1, 103)	186	73.2 (44.7, 126.5)	177
Iodine/creatinine ratio (μg/g)^a,b^	104 (66, 172)	190	119 (82, 186)	186	127 (86, 184)	177
Tg (μg/L)^a^	21 (13, 33)	185	19 (12, 30)	183	23 (13, 33)	179
TSH (mU/L)^a^	1.3 (0.1, 4.2)	190	1.8 (0.4, 4.0)	185	2.0 (0.4, 5.4)	179
fT4 (pmol/L)^a^	15.1 (1.8)	190	12.6 (1.4)	185	11.2 (1.4)	179

^a^ Values are expressed as median (25th, 75th percentile) for iodine concentration, iodine-to-creatinine ratio, and Tg; fT4 values are expressed as mean (*SD*); TSH and gestational week are expressed as median (min, max).

^b^ Values differ from reference 13, as women with thyroid dysfunction are excluded here.

TPOAb, thyroid peroxidase antibodies; TgAb, thyroglobulin antibodies; Tg, thyroglobulin; TSH, thyrotropin; fT4, free thyroxine.

The median Tg concentration was 21 μg/L in the first trimester (*n* = 185), 19 μg/L in the second trimester (*n* = 183), and 23 μg/L in the third trimester (*n* = 179; Table 1). In the first, second, and third trimesters, 29 (15.7%), 16 (8.7%), and 33 (18.4%) women, respectively, had a high Tg, defined as a concentration >40 μg/L according to the reference range suggested in studies of schoolchildren and adults (9).

A linear mixed model was constructed to explore the effect of iodine status and gestational week on both TSH and Tg while controlling for season, maternal age, smoking status, BMI, and ethnicity. The interaction term between the iodine variable (<150 and ≥150 μg/g) and gestational week was significant in both the TSH (*p* = 0.010) and Tg (*p* = 0.012) models. To interpret the effect of the iodine-status group on Tg and TSH concentration, estimated marginal means were reported while holding the continuous interacting variable, gestational week, at the mean. Tg was significantly (*p* < 0.001) higher in the <150 μg/g group compared with the ≥150 μg/g group (estimated marginal mean 18 vs. 16 μg/L; *p* < 0.001). By contrast, there was no significant difference in TSH concentration between the <150 and ≥150 μg/g groups at the mean gestational week (estimated marginal mean TSH 1.49 vs. 1.55 mIU/L; *p* = 0.27).

**FIG. 1.**
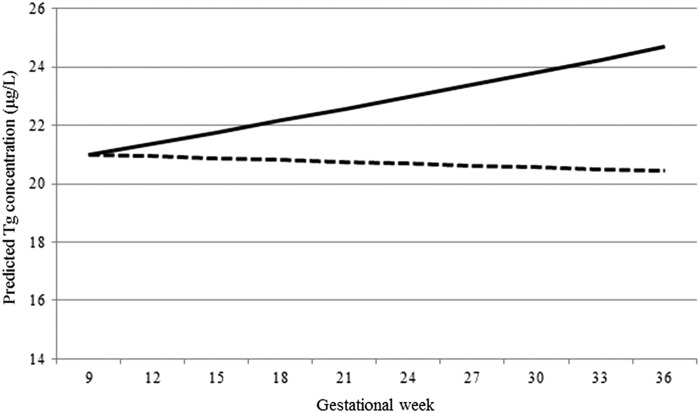
Change in thyroglobulin (Tg) throughout gestation according to iodine-status group. Predicted values for Tg based on median at baseline and geometric mean ratio for a one-week increase in the <150 μg/g group (solid line) and the ≥150 μg/g group (dashed line). Results are from a linear mixed model (on log-transformed data), controlling for the effects of season (winter/summer), body mass index (<25 vs. ≥25 kg/m^2^), smoking status (never vs. ex-smoker), ethnicity (Caucasian vs. other), and maternal age. The interaction between iodine status and gestational week was significant (*p* = 0.012).

The interaction between gestational week and the dichotomized iodine variable meant that there was no significant increase in Tg with advancing pregnancy in the ≥150 μg/g group, whereas there was an increase in Tg in the <150 μg/g group (Fig. 1 and Table 2). Tg was higher in the <150 μg/g group than it was in the ≥150 μg/g at each time point of gestation, but the difference between the groups was greater in the later stages of pregnancy (Table 3). TSH increased with advancing gestation in both iodine-status groups, but the increase was greater in the <150 μg/g group than it was in the 150 μg/g group (Table 2).

**Table 2.** Linear Mixed Model Exploring the Interaction Between Iodine Status and Week of Gestation on TSH and Tg Concentrations

		*TSH*		*Tg*	
	*Urinary iodine-to-creatinine ratio group*	*Geometric mean ratio [CI]*^a^	p*-Value for interaction*^b^	*Geometric mean ratio [CI]*^a^	p*-Value for interaction*^b^
One week increase in gestation	<150 μg/g	1.020 [1.016–1.023]	0.010	1.006 [1.003–1.008]	0.012
	≥150 μg/g	1.011 [1.001–1.021]		0.999 [0.992–1.007]	

^a^ Exponential of β from linear mixed model, controlling for the effects of season (winter/summer), body mass index (<25 vs. ≥25 kg/m^2^), smoking status (never vs. ex-smoker), ethnicity (Caucasian vs. other), and maternal age.

^b^ *p*-Value for the difference in slope (<150 vs. ≥150 μg/g).

CI, confidence interval.

**Table 3.** Tg Concentration in the First, Second, and Third Trimesters of Pregnancy According to Iodine Group

		*Tg concentration (μg/L)*^a^		
		*12 weeks*	*20 weeks*	*35 weeks*
Urinary iodine-to-creatinine ratio group	<150 μg/g	17 [14–21]	18 [15–22]	20 [16–24]
	≥150 μg/g	16 [13–20]	16 [13–20]	16 [13–20]
*p*-Value^b^		0.056	<0.001	<0.001

^a^ Data are estimated marginal mean [CI]. Estimated marginal means are calculated from the linear mixed-model that controlled for the effects of season (winter/summer), body mass index (<25 vs. ≥25 kg/m^2^), smoking status (never vs. ex-smoker), ethnicity (Caucasian vs. other), and maternal age. It also included an interaction term between iodine group and gestational week. The estimated marginal means were computed for these time points to reflect the study design.

^b^ *p*-Value comparing the Tg concentration in the <150 and ≥150 μg/g iodine group at each time point.

**FIG. 2.**
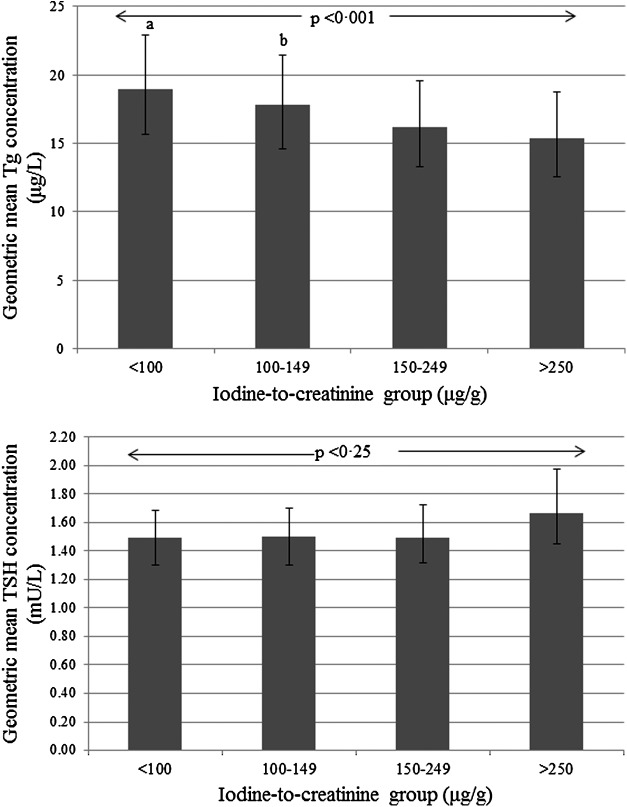
Adjusted geometric mean TSH and Tg concentration by iodine status (as iodine-to-creatinine ratio, μg/g). The error bars are the confidence intervals of the geometric mean. Results are computed by back transformation of estimated marginal means from a linear mixed model (on log-transformed data), controlling for the effects of gestational week, season (winter/summer), body mass index (<25 vs. ≥25 kg/m^2^), smoking status (never vs. ex-smoker), ethnicity (Caucasian vs. other), and maternal age. ^a^Significantly higher than the 150–249 μg/g group (*p* < 0.001) and the ≥250 μg/g group (*p* < 0.001). ^b^Significantly higher than the 150–249 μg/g group (*p* = 0.005) and the ≥250 μg/g group (*p* = 0.002).

When the four iodine-status groups (<100, 100–149, 150–249, and ≥250 μg/g) were explored, there was no significant interaction between the iodine variable and gestational week on either TSH (*p* = 0.07) or Tg (*p* = 0.054) concentration (likely because of reduced power with a smaller sample size in each group). Therefore, only main effects are reported. While there was no difference in TSH concentration between the four groups (*p* = 0.25), Tg concentrations differed significantly (*p* < 0.001; Fig. 2). Women in the <100 μg/g and 100–149 μg/g groups had significantly higher Tg concentrations than those in the 150–249 and the ≥250 μg/g groups (Fig. 2). Against the reference group of 150–249 μg/g, the geometric mean ratio of the Tg concentration was 17% higher in the <100 μg/g group (1.17, [CI 1.10–1.25]) and 10% higher in the 100–149 μg/g group (1.10 [CI 1.03–1.17]). There was no significant difference between the >250 μg/g group and the 150–249 μg/g group (Supplementary Table S1; Supplementary Data are available online at www.liebertpub.com/thy).

The study explored whether dietary intake of milk was associated with Tg concentration at 12 weeks of gestation (i.e., when the FFQ was completed). An inverse relationship was found between Tg concentration and milk intake, such that median Tg was lowest in the top milk-intake group (i.e., 24, 21, and 18 μg/L in the <140 mL, 140–280 mL, and >280 mL/day groups, respectively; *p* = 0.13). The difference between those in the <140 mL group and those in the >280 mL group was significant at the 90% level (*p* = 0.06). Milk consumption was significantly inversely associated with the percentage of women with high Tg (*p* = 0.02), with 28.3%, 12.2%, and 8.9% of women in the <140 mL, 140–280 mL, and >280 mL groups having a Tg concentration >40 μg/L. There was no relationship between milk intake and TSH (data not shown).

## Discussion

In line with the hypothesis, a negative association was found between iodine status (as measured by the iodine-to-creatinine ratio) and serum Tg concentration. Tg was higher in the group with an iodine-to-creatinine ratio <150 μg/g than it was in the group with a ratio >150 μg/g. Furthermore, when the <150 μg/g and the ≥150 μg/g groups were subdivided, there was a trend for increasing Tg concentration across the four iodine-status groups, with the highest Tg concentration being in those with an iodine-to-creatinine ratio <100 μg/g. These results suggest that Tg may be used as a functional marker of iodine deficiency in a mildly-to-moderately iodine deficient population, and is a notably better marker than TSH.

The results also demonstrate that the effect of advancing gestation on TSH and Tg differs between iodine-sufficient and iodine-deficient women, as was hypothesized. The study shows that in the <150 μg/g group, the increase in TSH during pregnancy was greater than it was in the ≥150 μg/g group. Furthermore, Tg concentration increased throughout pregnancy in the <150 μg/g group, whereas there was no significant change in the ≥150 μg/g group. The difference in Tg concentration between the iodine groups was greatest in later pregnancy. Taken together, these results suggest that even mild-to-moderate iodine deficiency during pregnancy stresses the thyroid. It should be remembered here that thyroid-antibody positive women were excluded from the analysis, which means that the Tg concentration was not influenced by autoimmune destruction of thyroid cells.

The increase in TSH throughout gestation in both iodine-status groups reflects the well-known physiological change (15). In the first trimester, TSH is suppressed as a result of the transient effect of human chorionic gonadotropin (hCG). TSH then increases in the second and third trimesters (15). However, the finding of a greater increase in TSH in the <150 μg/g group may reflect hyperstimulation of the thyroid under conditions of iodine deficiency. The increase in Tg concentration in the iodine-deficient women suggests that thyroid volume is increasing as pregnancy advances. Initially (first trimester), this is likely to be a result of hyperstimulation of the thyroid by hCG, but the later increase probably reflects thyroidal adaptation to the low dietary iodine supply. It has previously been suggested that an increase in thyroid size in pregnancy may be a risk factor for impaired supply of thyroid hormones to the fetus and that the increased size may not completely regress after pregnancy, leading to later thyroid dysfunction in the mother (22).

Randomized controlled trials (RCTs) in pregnant women from regions of mild-to-moderate iodine deficiency have found that iodine supplementation can prevent the increase in thyroid size and Tg concentration seen in the control groups with advancing pregnancy (23–25). The present results support the findings from those RCTs, as no increase in Tg was found in the iodine-sufficient group (i.e., those with an iodine-to-creatinine ratio ≥150 μg**/**g).

Tg has shown promise as an iodine-status marker, and has been used successfully, along with UIC, in schoolchildren from different countries (12) and in adults in China (26). In a recent RCT, Tg was shown to be responsive to improved iodine status in adults following 24 weeks of iodine supplementation (27). However, the potential for Tg to be a longer-term biomarker of iodine status in pregnant women has seldom been explored, and this is the first such study in British women.

A strength of this study is that it used repeated measures for both Tg and urinary iodine excretion in pregnant women. Although two other studies also had repeated measures, they either did not report the relationship between urinary iodine status and Tg (28), or they only used simple cross-sectional analysis at each trimester (29). Therefore, to the authors' knowledge, this is the first study to use a repeated-measures analysis to relate iodine status to Tg concentration and to explore the change in Tg concentration during pregnancy in relation to urinary iodine excretion. Other studies in pregnancy have measured iodine status and Tg at one time point and have not found an association with either UIC (30,31) (possibly as a result of small sample sizes) or with 24 h urine excretion (32). However, two studies found a significant negative association between UIC and Tg that was stronger than the association between UIC and TSH (20,29). These studies support the present findings. A recent review of Tg found the median Tg to be ≥13 μg**/**L in the majority of studies of pregnant women from iodine-deficient areas (27), as was the case in our study of mildly to moderately deficient women where the median Tg concentration was 21, 19, and 23 μg**/**L in trimesters one, two, and three, respectively. There is evidence, both from schoolchildren (12) and pregnant women (33), that the relationship between UIC and Tg is U-shaped. However, there was no evidence of non-linearity in this study, probably because most women were deficient, and it was therefore not possible to explore the effects of iodine excess.

Low intake of milk, the major source of iodine in the United Kingdom, was found to be associated with a higher Tg concentration, suggesting that low iodine intake, either pre-pregnancy or early in pregnancy (the FFQ reflected intake over the previous 12 months), results in increased thyroid size. This finding is similar to that from a study in Denmark that found an association between a low iodine index (based on milk and fish intake) and higher Tg concentration in adults (males and females) (34). Furthermore, Danish pregnant mothers who took an iodine-containing supplement had significantly lower Tg levels at term (35).

The significant interaction found between iodine status and gestational week suggests that simple cross-sectional analysis may not reveal the relationship between iodine and TSH because there may be a lag between inadequate iodine intake and evidence of thyroid dysfunction. Indeed, when women were dichotomized (<150 and ≥150 μg/g) according to their iodine-to-creatinine ratio at 12 weeks, although there was no difference in TSH concentration, which was also measured at 12 weeks, TSH was higher at 20 (*p* = 0.008) and 35 (*p* = 0.06) weeks of gestation in the women who were in the <150 μg/g group at 12 weeks. This suggests a lag effect of low iodine status on serum TSH, and might explain why previous studies in pregnant women in iodine-deficient regions that have only examined relationships cross-sectionally have not found significant associations between iodine status and TSH (6,30,36–40).

This study has a number of limitations. First, the women were recruited as part of a trial, and therefore may be of higher socioeconomic and educational status than the general population. It has previously been shown that maternal education is positively associated with iodine-to-creatinine ratio in pregnant women in this and in another cohort (7,13). Second, the study was conducted in a region of mild-to-moderate iodine deficiency, reducing the ability to explore the relationship between iodine and Tg across the full range of iodine status. Third, women with thyroid antibodies were excluded, and therefore the findings relate to “healthy” pregnant women without overt (or subclinical) thyroid dysfunction. Fourth, thyroid volume was not assessed by ultrasound, hence it was not possible to correlate higher Tg concentration with thyroid size.

In conclusion, Tg shows promise as a long-term marker of iodine status in pregnant women. A method for measuring Tg in dried blood spots (DBS) has recently been developed in samples from pregnant women that may be useful in future studies (41). However, the inter-assay variability (even if calibrated to CRM-457) makes the determination of a general clinical cutoff for Tg concentration challenging. Tg measurement is invasive and an additional expense, but this study suggests that urinary iodine excretion and Tg may be complementary measures of iodine status and may give a better picture of status than either measure alone. Future studies should therefore consider measurement of Tg concentration (concurrently with TgAb, as 10% of women in this study were TgAb-positive, which tended to decrease Tg) in addition to urinary iodine-to-creatinine ratio when assessing the iodine status of pregnant women.

## Supplementary Material

Supplemental data
