# Supplementary material for: Thyroglobulin as a Functional Biomarker of Iodine Status in a Cohort Study of Pregnant Women in the United Kingdom
Source: Thyroid. 2017 Mar 1;27(3):426–33. doi: 10.1089/thy.2016.0322 (PMC5337401; doi:10.1089/thy.2016.0322)
Supplement: Supplemental data [file Supp_Table1.pdf]

Supplementary Data

SUPPLEMENTARY TABLE S1. EFFECT OF IODINE STATUS (IN FOUR GROUPS) AND GESTATIONAL WEEK ON TSH AND TG DURING PREGNANCY—RESULTS FROM A MAIN-EFFECTS LINEAR MIXED MODEL

		TSH (mIU/L)		*Tg (μg/L)*	
		Geometric mean ratio [CI][a]	p-*Value*	*Geometric mean ratio [CI]*[a]	p-*Value*
Urinary iodine-to-creatinine ratio group, μg/g	<100 vs. 150–249	1.00 [0.92–1.09]	0.25	1.17 [1.10–1.25]	<0.001
	100–149 vs. 150–249	1.00 [0.93–1.09]		1.10 [1.03–1.17]	
	≥250 vs. 150–249	1.11 [1.00–1.24]		0.95 [0.88–1.03]	
Gestational week of sample		1.016 [1.013–1.020]	<0.001	1.004 [1.002–1.006]	<0.001

Geometric mean ratios relate to Tg in each iodine category compared to Tg in the reference group of 150–249 μg/g.
[a]Exponential of β from the linear mixed model (controlling for the main effects of season [winter/summer], BMI [<25 vs. ≥25 kg/m^2], smoking status [never vs. ex-smoker], ethnicity [Caucasian vs. other], and maternal age).
